# Enhancing Microbiome Research through Genome-Scale Metabolic Modeling

**DOI:** 10.1128/mSystems.00599-21

**Published:** 2021-12-14

**Authors:** Nana Y. D. Ankrah, David B. Bernstein, Matthew Biggs, Maureen Carey, Melinda Engevik, Beatriz García-Jiménez, Meiyappan Lakshmanan, Alan R. Pacheco, Snorre Sulheim, Gregory L. Medlock

**Affiliations:** a State University of New York at Plattsburgh, Plattsburgh, New York, USA; b University of California, Berkeley, California, USA; c Sestina Bio, Pleasanton, California, USA; d University of Virginia, Charlottesville, Virginia, USA; e Medical University of South Carolina, Charleston, South Carolina, USA; f Biome Makers, Inc., West Sacramento, California, USA; g Bioprocessing Technology Institute, Agency for Science, Technology and Research (A*STAR), Singapore; h Bioinformatics Institute, Agency for Science, Technology and Research (A*STAR), Singapore; i ETH Zurich, Zurich, Switzerland; j SINTEF Industry, Trondheim, Norway; k Vedanta Biosciences, Cambridge, Massachusetts, USA; California State University, Monterey Bay

**Keywords:** microbiome, metabolic modeling, metabolism

## Abstract

Construction and analysis of genome-scale metabolic models (GEMs) is a well-established systems biology approach that can be used to predict metabolic and growth phenotypes. The ability of GEMs to produce mechanistic insight into microbial ecological processes makes them appealing tools that can open a range of exciting opportunities in microbiome research. Here, we briefly outline these opportunities, present current rate-limiting challenges for the trustworthy application of GEMs to microbiome research, and suggest approaches for moving the field forward.

## PERSPECTIVE

## OPPORTUNITIES

Genome-scale metabolic models (GEMs) are mathematical representations of the chemical reactions that can be carried out by an organism. In their simplest form, they encode the quantities of reactants and products consumed or produced in chemical reactions. As such, GEMs can simulate the growth of organisms in diverse environmental contexts through approaches such as flux balance analysis (1) and have become essential tools for generating testable hypotheses about their metabolic functions. This combination of versatility and mechanistic insight allows GEMs to be readily extended to investigate the roles of individual species in complex microbial assemblages (2–4). In particular, a collection of GEMs can predict the metabolic interactions that can emerge between members of microbial communities by calculating the flow of metabolites through each organism and its environment (5). As such, GEM simulations can also predict environmental modifications that can occur as a result of metabolite secretion and utilization by community members (6). GEMs can be used with experimental or observational data (referred to as “context-specific modeling”) (7) and in time- and spatially resolved simulations with single or multiple organisms (8, 9). These approaches can all be extended to multicompartment models to study host-microbe interactions by simultaneously modeling host and microbial metabolism. In particular, such frameworks have been used to predict microbiome responses to interventions that are challenging to represent experimentally (e.g., dynamic environments, invading species in sensitive ecosystems, clinical scenarios) (10, 11). GEMs also provide opportunities to generate insights from microbiome multiomics, i.e., metagenomics, metatranscriptomics, metaproteomics, and/or metabolomics data (12–15).

Most microbiome studies employing GEMs use them to explicitly model a microbiome (i.e., a single simulation captures metabolism of the entire microbiome). Alternatively, the physiological factors represented by a GEM (e.g., biochemical capabilities, genome annotations) or the results of simulations with individual organisms’ GEMs (e.g., simulated nutrient preferences, simulated metabolite production) can be used outside the context of microbiome-wide *in silico* simulations to evaluate new hypotheses with metagenomics and metabolomics data (12, 16, 17). For example, rather than using GEMs to simulate fluxes in a community, simulations with GEMs for individual organisms can be used to predict metabolic traits for members of a community. Statistical analyses (e.g., regression, classification) can then be performed using these traits as input to predict environmental observations (e.g., nutrient abundances, plant yields, clinical outcomes) (17). These applications, as well as their use across a variety of systems and temporospatial scales, have been thoroughly reviewed (18–21).

## CHALLENGES

**FIG 1:**
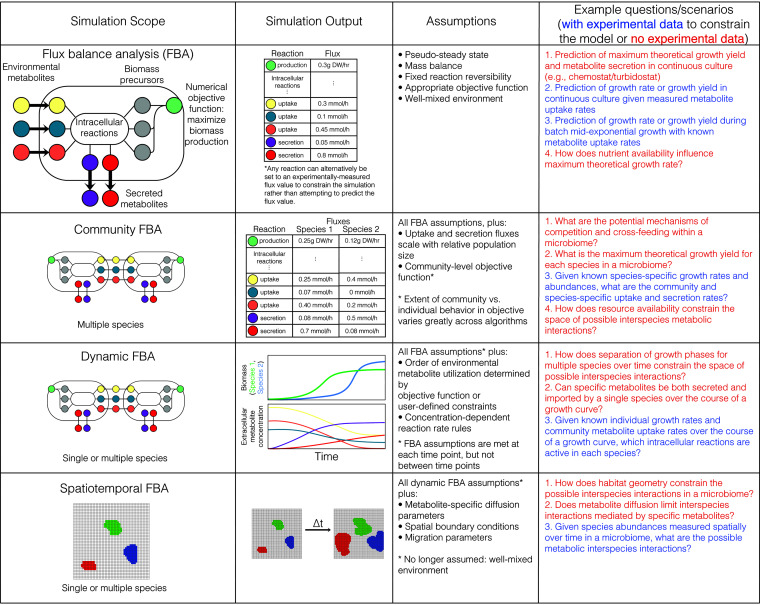
Choice of microbiome modeling scope with genome-scale metabolic models (GEMs). Row 1 shows analysis of individual species (alone or within a microbiome) at a single time point with flux balance analysis (FBA). FBA can also be used for bag-of-genes models of a microbiota, which include all metabolic functions of the microbiota within a single compartment. Row 2 shows simulation of multiple species at a single time point in a community context with community FBA (where “Community FBA” is inclusive of all nondynamic FBA-based methods employing GEMs for multiple organisms in a shared extracellular compartment). Row 3 shows dynamic FBA, wherein metabolic fluxes and growth are simulated for a community with objective functions at the individual organism level. In dynamic FBA, multiple iterations of FBA are performed to introduce a temporal dimension. Community dynamics emerge in dynamic FBA through the shared extracellular compartment. Row four shows spatiotemporal FBA, which introduces spatial dimensions within a dynamic FBA framework to account for diffusion (or environmentally induced mixing) of metabolites and microbial biomass. Example simulation output and a nonexhaustive list of critical assumptions are listed for each scope. Examples of appropriate scenarios and questions are shown for each scope, with examples showing how introducing experimental data to constrain GEMs (text in blue) can increase the specificity of the simulation output at each scope relative to scenarios where little or no experimental data are used to constrain GEMs (text in red). Note that the following terms are not interchangeable: growth rate (growth per unit time), growth yield (growth per unit substrate uptake), and maximum theoretical growth yield (maximum growth per unit substrate, determined by stoichiometry and reversibility alone).

To ensure the reliability of GEM predictions, it is crucial to establish the appropriate context for their application to microbial communities (22). First, one must consider that the application of GEMs is generally limited to metabolic interactions, while other factors key to microbial community dynamics (e.g., gene regulation, expression, and protein localization; pH’ temperature; antibiotics; and quorum sensing) are only accounted for in specific modeling extensions (8, 23–33). Second, one should select a simulation scope according to the experimental question raised, preferred simulation output, appropriate assumptions, and available data (Fig. 1).

The application of flux balance analysis (FBA) to microbial communities necessitates the study of many nonmodel organisms, requiring either reconstruction of strain-specific GEMs for a particular microbiome or utilizing “bag-of-genes” models in which all microbial metabolic capabilities are combined into one network (34–37) (Fig. 1, row 1). While model reconstruction has traditionally been a major bottleneck, recently developed GEM reconstruction pipelines and curated resources are eliminating many of the challenges associated with throughput and model quality (32, 36, 38–40). A remaining challenge, however, is to account for model structural uncertainty introduced through gene annotation, gene-to-reaction mapping, environment specification, and biomass composition. In combination with degeneracy in FBA solutions, this accumulates to a total uncertainty associated with model predictions that is difficult to quantify (41). Ensemble modeling is a promising approach for representing this uncertainty, but challenges remain in scaling these approaches to address heterogeneous sources of uncertainty at the microbiome level (42, 43).

Scaling the scope of GEM simulations to communities (Fig. 1, row 2) results in a number of additional sources of uncertainty and technical challenges. First, different chemical and reaction namespaces between disparate data sets and models can hinder the combination of GEMs from different sources (44, 45). Additionally, community FBA methods require the formulation of a community-level objective that both draws from biological rationales and balances the trade-off between community-level and individual objectives, making it difficult to accurately predict phenotypes for complex microbiomes (21, 46).

Instead of defining a community-level objective, microbial interactions mediated via the environment can be simulated through dynamic FBA (Fig. 1, row 3), a technique that allows community dynamics to emerge from individual-level objectives. However, dynamic FBA requires additional model parameters (e.g., metabolite- and organism-specific uptake kinetics) that are challenging to estimate for a complex microbiome. Spatial dynamics such as diffusion can be combined with dynamic FBA through more advanced modeling frameworks (9, 47), but this also introduces parameters which are difficult to estimate (Fig. 1, row 4). Increasing the time scale of dynamic microbiome simulations is possible but complicates the underlying mechanisms as metabolic adaptation and evolution play into eco-evolutionary dynamics (48, 49).

As models scale up to represent dynamics at these higher levels, it is important to understand the propagation of the uncertainties mentioned above to ensure robust simulation frameworks. Efforts toward modeling microbiomes with individual-organism resolution need to consider the current state of accuracy and uncertainty in each constituent GEM, as well as the sensitivity of the phenotype of interest to this uncertainty. In the simplest case, we may consider an *in silico* study of two bacterial strains during steady-state growth with glucose as a sole carbon source. Here, the goal of the study would be to predict whether a coculture of the two organisms will increase biomass yield for either individual organism. If the coculture simulation accounts for uncertainty, the researchers can base their degree of trust in the simulation on how the predictions are distributed. The researchers can also use uncertainty in monoculture simulations for any functions related to the coculture outcomes (growth yield in the fresh medium, metabolite secretion, and uptake and metabolism of putatively cross-fed metabolites) to identify functions in individual GEMs that should be curated to reduce uncertainty (and potentially improve accuracy) of the coculture simulations.

Phenotyping individual organisms in high throughput is a promising approach toward improving GEM curation; however, it is not always feasible (e.g., obligate intracellular organisms) or may be experimentally challenging to set up (e.g., organisms which have no known, chemically defined culture conditions) (36, 50). Furthermore, when studies are scaled to the complexity of most natural microbiomes, it becomes infeasible to test the performance of each individual organism in well-controlled monocultures. As we attempt to make use of increasingly complex models of microbiomes, systematic curation strategies will need to be coupled with expert intuition to establish the appropriate level of detail that is coarse-grained enough to be robust to parameter uncertainty and stochastic community dynamics (51–53) yet detailed enough to capture important processes. Researchers should keep this in mind when selecting the appropriate simulation scope for the application of GEMs to microbial communities and should try to balance the opportunities and challenges of each approach. We discuss the implications of these challenges for scientific funding priorities in the following section.

## MOVING FORWARD

The potential of GEMs to advance microbiome science is founded upon a rapidly growing body of genomic (and, increasingly, transcriptomic and metabolomic) data, which has already enabled researchers and clinicians to explore the metabolic functions of microbiomes associated with diverse ecosystems—from soil and plants (54, 55), to insects (56), to mammals (36, 57). However, these advances have largely been driven by independent analyses by theoretical and empirically focused research groups. Thus, they have resulted in a fragmentary understanding of the mechanisms and impact of interspecies interactions on microbiome function. While many opportunities exist for two-way communication between theoretical and empirical microbiome research communities, several barriers prevent this potential from being realized.

**FIG 2:**
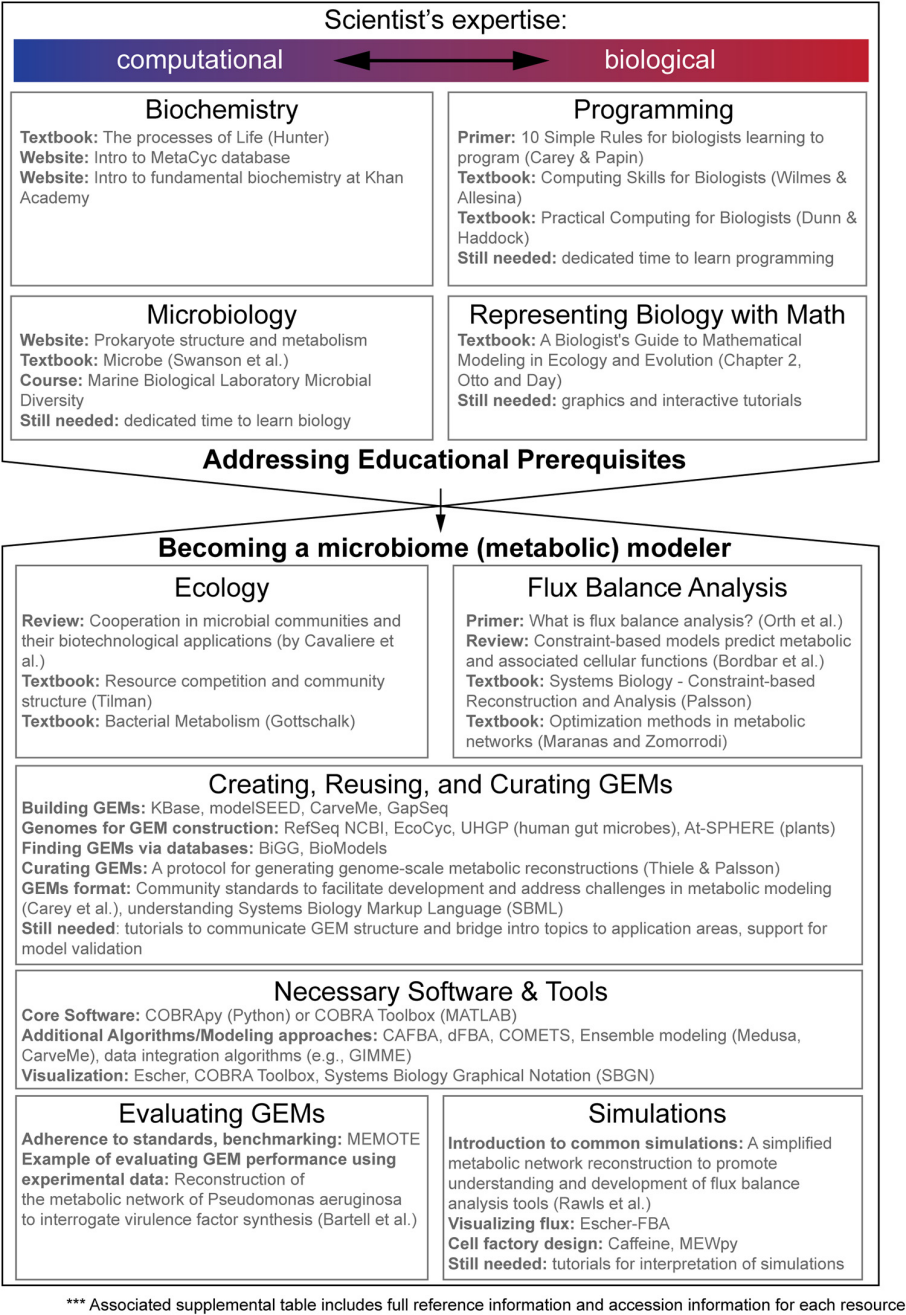
Opportunities for training and establishing curricula to prepare scientists to apply genome-scale metabolic modeling in microbiome studies. Each topic rectangle lists resources we feel are beneficial for scientists exploring genome-scale metabolic modeling of microbiomes. All resources are listed with accession information in Table S1. The top half includes prerequisite topics based on researcher background; those with more computational experience (left side) or more biological experience (right side) will have different knowledge gaps. The bottom half includes resources that should be helpful regardless of background. Content in “still needed” lines was identified by our authors as having strong deficiencies in available learning material.

In order to better understand these barriers, we conducted a community survey of microbiome scientists (Fig. S1, Table S2). This survey revealed that, while over 70% of empirical researchers expressed an interest in using metabolic modeling, a lack of computational expertise and concern about the accuracy of predictions has prevented them from integrating models into their work. Based on these responses, we advocate for increased accessibility of modeling techniques and more transparent communication and interpretation of simulation results. To this end, we have compiled existing resources to initiate microbiome scientists—from a range of backgrounds—to use genome-scale modeling to address questions about the microbiome (Fig. 2). Importantly, we have highlighted resource gaps that must be filled to maximize the accessibility of the modeling field and facilitate cross talk between theoretical and empirical researchers. Such resources will enable microbiome scientists to determine the appropriateness of applying this modeling framework to their research questions.

10.1128/mSystems.00599-21.1 FIG S1 Graphical display of community survey results. The community survey link was shared via twitter and multiple forums for software related to constraint-based reconstruction and analysis of metabolic networks. All individual survey responses, including responses to additional free-response survey questions, are included in Table S2. Download FIG S1, TIF file, 2.9 MB. Copyright © 2021 Ankrah et al. 2021 Ankrah et al. https://creativecommons.org/licenses/by/4.0/ This content is distributed under the terms of the Creative Commons Attribution 4.0 International license.

10.1128/mSystems.00599-21.2 TABLE S1 Compiled educational resources, with accession information, in table format for resources shown in Fig. 2. Resources are organized with the same section headers as in Fig. 2. Download Table S1, XLSX file, 0.01 MB. Copyright © 2021 Ankrah et al. 2021 Ankrah et al. https://creativecommons.org/licenses/by/4.0/ This content is distributed under the terms of the Creative Commons Attribution 4.0 International license.

10.1128/mSystems.00599-21.3 TABLE S2 All individual responses to the community survey. Option-based question responses are summarized in Fig. S1. Download Table S2, XLSX file, 0.02 MB. Copyright © 2021 Ankrah et al. 2021 Ankrah et al. https://creativecommons.org/licenses/by/4.0/ This content is distributed under the terms of the Creative Commons Attribution 4.0 International license.

In addition to the points raised by empirical researchers in our survey, participants with modeling experience echoed our concerns regarding the lack of universal approaches to verify model robustness and evaluate predictions. This challenge is especially important in microbiome science, given the potential for error propagation associated with scaling up models to the enormous diversity of organisms found in many microbiomes. We advocate for improving model trustworthiness via transparent modeling practices (construction, curation, and validation) and the integration of uncertainty in predictions. Just as community efforts have led to advances and standardization in GEM validation (58), similar endeavors are needed to ensure transfer of knowledge across studies, both in specific scientific application areas and in the practice of GEM reconstruction. These efforts should focus on ensuring that GEM construction and curation processes are transparent and modifiable (59).

We close with specific recommendations for the microbiome modeling community and funding agencies to address the challenges and capture the opportunities we have highlighted.

• First, the modeling community should create new mechanisms for evaluating GEM construction and simulation methodology. Specifically, we recommend establishing competitive challenges with objective assessments (60, 61). As an example, the Critical Assessment of protein Structure Prediction (CASP) has been funded by the NIH for over 20 years at about $500,000 per year and has yielded a variety of useful methods for predicting protein structure.
• Second, funding agencies should establish mechanisms and proposal expectations that incentivize development, maintenance, and improvement of modeling methods and tools. As in bioinformatics tool development (62), genome-scale metabolic modeling methods are nearly exclusively developed *ad hoc* through grants focused on application rather than development of methods. We see two divergent paths to foster collaborative microbiome studies enabled by metabolic modeling:
  o Increase budget limits for proposals involving collaborating specialist groups.
  o Reduce expectations of interdisciplinarity in grant proposals. As nearly all modelers are interested in collaborating with experimentalists, providing funding stability which is not contingent on a collaboration will reduce grant writing burden and administrative complexity and encourage transparent communication of modeling results between collaborators. Experimentalists will also feel more comfortable only pursuing modeling approaches that are likely to add value to their work.

Early career researchers, who often choose to specialize to demonstrate productivity and compete for funding, will benefit most from reduced collaborative expectations at the grant proposal review stage and a culture of transparent modeling. Efforts to increase methodological transparency in the metabolic modeling field will also benefit early career researchers by lowering barriers to entry for researchers from diverse disciplines and backgrounds. The training resources enabled through transparency will increase access to modeling tools and allow researchers without access to costly experimental technologies greater opportunity to do science. These aspects are particularly relevant to researchers using GEMs within the microbiome field, which is already incredibly interdisciplinary. Ultimately, the challenges associated with applying GEMs to the microbiome field emphasize fundamental opportunities for growth of the genome-scale metabolic modeling field.

## Supplementary Material

Reviewer comments

## References

[1] Orth JD, Thiele I, Palsson BØ. 2010. What is flux balance analysis? Nat Biotechnol 28:245–248. doi:10.1038/nbt.1614. 20212490 PMC3108565

[2] Varma A, Palsson BO. 1994. Stoichiometric flux balance models quantitatively predict growth and metabolic by-product secretion in wild-type Escherichia coli W3110. Appl Environ Microbiol 60:3724–3731. doi:10.1128/aem.60.10.3724-3731.1994. 7986045 PMC201879

[3] Fang X, Lloyd CJ, Palsson BO. 2020. Reconstructing organisms in silico: genome-scale models and their emerging applications. Nat Rev Microbiol 18:731–743. doi:10.1038/s41579-020-00440-4. 32958892 PMC7981288

[4] McCloskey D, Palsson BØ, Feist AM. 2013. Basic and applied uses of genome-scale metabolic network reconstructions of Escherichia coli. Mol Syst Biol 9:661. doi:10.1038/msb.2013.18. 23632383 PMC3658273

[5] Hutchinson GE. 1957. Concluding remarks. Cold Spring Harbor Symp Quant Biol 22:415–427. doi:10.1101/SQB.1957.022.01.039.

[6] Pacheco AR, Moel M, Segrè D. 2019. Costless metabolic secretions as drivers of interspecies interactions in microbial ecosystems. Nat Commun 10:103. doi:10.1038/s41467-018-07946-9. 30626871 PMC6327061

[7] Blazier AS, Papin JA. 2012. Integration of expression data in genome-scale metabolic network reconstructions. Front Physiol 3:299. doi:10.3389/fphys.2012.00299. 22934050 PMC3429070

[8] Harcombe WR, Riehl WJ, Dukovski I, Granger BR, Betts A, Lang AH, Bonilla G, Kar A, Leiby N, Mehta P, Marx CJ, Segrè D. 2014. Metabolic resource allocation in individual microbes determines ecosystem interactions and spatial dynamics. Cell Rep 7:1104–1115. doi:10.1016/j.celrep.2014.03.070. 24794435 PMC4097880

[9] Bauer E, Zimmermann J, Baldini F, Thiele I, Kaleta C. 2017. BacArena: individual-based metabolic modeling of heterogeneous microbes in complex communities. PLoS Comput Biol 13:e1005544. doi:10.1371/journal.pcbi.1005544. 28531184 PMC5460873

[10] García-Jiménez B, Carrasco J, Medina J, Wilkinson MD. 2020. Dynamic simulations of microbial communities under perturbations: opportunities for microbiome engineering. Res Square

[11] Geng J, Ji B, Li G, López-Isunza F, Nielsen J. 2021. CODY enables quantitatively spatiotemporal predictions on in vivo gut microbial variability induced by diet intervention. Proc Natl Acad Sci U S A 118:e2019336118. doi:10.1073/pnas.2019336118. 33753486 PMC8020746

[12] Shoaie S, Ghaffari P, Kovatcheva-Datchary P, Mardinoglu A, Sen P, Pujos-Guillot E, de Wouters T, Juste C, Rizkalla S, Chilloux J, Hoyles L, Nicholson JK, Dore J, Dumas ME, Clement K, Bäckhed F, Nielsen J, MICRO-Obes Consortium. 2015. Quantifying diet-induced metabolic changes of the human gut microbiome. Cell Metab 22:320–331. doi:10.1016/j.cmet.2015.07.001. 26244934

[13] Sen P, Orešič M. 2019. Metabolic modeling of human gut microbiota on a genome scale: an overview. Metabolites 9:22. doi:10.3390/metabo9020022. PMC6410263 30695998

[14] Kumar M, Ji B, Zengler K, Nielsen J. 2019. Modelling approaches for studying the microbiome. Nat Microbiol 4:1253–1267. doi:10.1038/s41564-019-0491-9. 31337891

[15] Sung J, Hale V, Merkel AC, Kim P-J, Chia N. 2016. Metabolic modeling with Big Data and the gut microbiome. Appl Transl Genom 10:10–15. doi:10.1016/j.atg.2016.02.001. 27668170 PMC5025471

[16] Noecker C, Chiu H-C, McNally CP, Borenstein E. 2019. Defining and evaluating microbial contributions to metabolite variation in microbiome-metabolome association studies. mSystems 4:e00579-19. doi:10.1128/mSystems.00579-19. 31848305 PMC6918031

[17] Garza DR, van Verk MC, Huynen MA, Dutilh BE. 2018. Towards predicting the environmental metabolome from metagenomics with a mechanistic model. Nat Microbiol 3:456–460. doi:10.1038/s41564-018-0124-8. 29531366

[18] Jansma J, El Aidy S. 2021. Understanding the host-microbe interactions using metabolic modeling. Microbiome 9:16. doi:10.1186/s40168-020-00955-1. 33472685 PMC7819158

[19] Biggs MB, Medlock GL, Kolling GL, Papin JA. 2015. Metabolic network modeling of microbial communities. Wiley Interdiscip Rev Syst Biol Med 7:317–334. doi:10.1002/wsbm.1308. 26109480 PMC4575871

[20] Bauer E, Thiele I. 2018. From network analysis to functional metabolic modeling of the human gut microbiota. mSystems 3:e00209-17. doi:10.1128/mSystems.00209-17. 29600286 PMC5872302

[21] Dillard LR, Payne DD, Papin JA. 2021. Mechanistic models of microbial community metabolism. Mol Omics 17:365–375. doi:10.1039/D0MO00154F. 34125127 PMC8202304

[22] Erdemir A, Mulugeta L, Ku JP, Drach A, Horner M, Morrison TM, Peng GCY, Vadigepalli R, Lytton WW, Myers JG Jr. 2020. Credible practice of modeling and simulation in healthcare: ten rules from a multidisciplinary perspective. J Transl Med 18:369. doi:10.1186/s12967-020-02540-4. 32993675 PMC7526418

[23] Chandrasekaran S, Price ND. 2010. Probabilistic integrative modeling of genome-scale metabolic and regulatory networks in Escherichia coli and Mycobacterium tuberculosis. Proc Natl Acad Sci U S A 107:17845–17850. doi:10.1073/pnas.1005139107. 20876091 PMC2955152

[24] Goelzer A, Fromion V, Scorletti G. 2011. Cell design in bacteria as a convex optimization problem. Automatica 47:1210–1218. doi:10.1016/j.automatica.2011.02.038.

[25] Imam S, Schäuble S, Brooks AN, Baliga NS, Price ND. 2015. Data-driven integration of genome-scale regulatory and metabolic network models. Front Microbiol 6:409. doi:10.3389/fmicb.2015.00409. 25999934 PMC4419725

[26] O’Brien EJ, Palsson BO. 2015. Computing the functional proteome: recent progress and future prospects for genome-scale models. Curr Opin Biotechnol 34:125–134. doi:10.1016/j.copbio.2014.12.017. 25576845 PMC4495013

[27] Jensen PA. 2018. Coupling fluxes, enzymes, and regulation in genome-scale metabolic models. Methods Mol Biol 1716:337–351. doi:10.1007/978-1-4939-7528-0_15. 29222761

[28] Persi E, Duran-Frigola M, Damaghi M, Roush WR, Aloy P, Cleveland JL, Gillies RJ, Ruppin E. 2018. Systems analysis of intracellular pH vulnerabilities for cancer therapy. Nat Commun 9:2997. doi:10.1038/s41467-018-05261-x. 30065243 PMC6068141

[29] Österberg L, Domenzain I, Münch J, Nielsen J, Hohmann S, Cvijovic M. 2021. A novel yeast hybrid modeling framework integrating Boolean and enzyme-constrained networks enables exploration of the interplay between signaling and metabolism. PLoS Comput Biol 17:e1008891. doi:10.1371/journal.pcbi.1008891. 33836000 PMC8059808

[30] Goldberg AP, Szigeti B, Chew YH, Sekar JA, Roth YD, Karr JR. 2018. Emerging whole-cell modeling principles and methods. Curr Opin Biotechnol 51:97–102. doi:10.1016/j.copbio.2017.12.013. 29275251 PMC5997489

[31] Bulović A, Fischer S, Dinh M, Golib F, Liebermeister W, Poirier C, Tournier L, Klipp E, Fromion V, Goelzer A. 2019. Automated generation of bacterial resource allocation models. Metab Eng 55:12–22. doi:10.1016/j.ymben.2019.06.001. 31189086

[32] Mendoza SN, Olivier BG, Molenaar D, Teusink B. 2019. A systematic assessment of current genome-scale metabolic reconstruction tools. Genome Biol 20:158. doi:10.1186/s13059-019-1769-1. 31391098 PMC6685185

[33] Bekiaris PS, Klamt S. 2020. Automatic construction of metabolic models with enzyme constraints. BMC Bioinformatics 21:19. doi:10.1186/s12859-019-3329-9. 31937255 PMC6961255

[34] Frioux C, Singh D, Korcsmaros T, Hildebrand F. 2020. From bag-of-genes to bag-of-genomes: metabolic modelling of communities in the era of metagenome-assembled genomes. Comput Struct Biotechnol J 18:1722–1734. doi:10.1016/j.csbj.2020.06.028. 32670511 PMC7347713

[35] Diener C, Gibbons SM, Resendis-Antonio O. 2020. MICOM: metagenome-scale modeling to infer metabolic interactions in the gut microbiota. mSystems 5:e00606-19. doi:10.1128/mSystems.00606-19. 31964767 PMC6977071

[36] Magnúsdóttir S, Heinken A, Kutt L, Ravcheev DA, Bauer E, Noronha A, Greenhalgh K, Jäger C, Baginska J, Wilmes P, Fleming RMT, Thiele I. 2017. Generation of genome-scale metabolic reconstructions for 773 members of the human gut microbiota. Nat Biotechnol 35:81–89. doi:10.1038/nbt.3703. 27893703

[37] Greenblum S, Turnbaugh PJ, Borenstein E. 2012. Metagenomic systems biology of the human gut microbiome reveals topological shifts associated with obesity and inflammatory bowel disease. Proc Natl Acad Sci U S A 109:594–599. doi:10.1073/pnas.1116053109. 22184244 PMC3258644

[38] Machado D, Andrejev S, Tramontano M, Patil KR. 2018. Fast automated reconstruction of genome-scale metabolic models for microbial species and communities. Nucleic Acids Res 46:7542–7553. doi:10.1093/nar/gky537. 30192979 PMC6125623

[39] Zimmermann J, Kaleta C, Waschina S. 2021. gapseq: informed prediction of bacterial metabolic pathways and reconstruction of accurate metabolic models. Genome Biol 22:81. doi:10.1186/s13059-021-02295-1. 33691770 PMC7949252

[40] Heinken A, Acharya G, Ravcheev DA, Hertel J, Nyga M, Okpala OE, Hogan M, Magnúsdóttir S, Martinelli F, Preciat G, Edirisinghe JN, Henry CS, Fleming RMT, Thiele I. 2020. AGORA2: large scale reconstruction of the microbiome highlights wide-spread drug-metabolising capacities. bioRxiv doi:10.1101/2020.11.09.375451.

[41] Bernstein DB, Sulheim S, Almaas E, Segrè D. 2021. Addressing uncertainty in genome-scale metabolic model reconstruction and analysis. Genome Biol 22:64. doi:10.1186/s13059-021-02289-z. 33602294 PMC7890832

[42] Biggs MB, Papin JA. 2017. Managing uncertainty in metabolic network structure and improving predictions using EnsembleFBA. PLoS Comput Biol 13:e1005413. doi:10.1371/journal.pcbi.1005413. 28263984 PMC5358886

[43] Medlock GL, Papin JA. 2020. Guiding the refinement of biochemical knowledgebases with ensembles of metabolic networks and machine learning. Cell Syst 10:109–119.e3. doi:10.1016/j.cels.2019.11.006. 31926940 PMC6975163

[44] Pham N, van Heck RGA, van Dam JCJ, Schaap PJ, Saccenti E, Suarez-Diez M. 2019. Consistency, inconsistency, and ambiguity of metabolite names in biochemical databases used for genome-scale metabolic modelling. Metabolites 9:28. doi:10.3390/metabo9020028. PMC6409771 30736318

[45] Moretti S, Tran VDT, Mehl F, Ibberson M, Pagni M. 2021. MetaNetX/MNXref: unified namespace for metabolites and biochemical reactions in the context of metabolic models. Nucleic Acids Res 49:D570–D574. doi:10.1093/nar/gkaa992. 33156326 PMC7778905

[46] Colarusso AV, Goodchild-Michelman I, Rayle M, Zomorrodi AR. 2021. Computational modeling of metabolism in microbial communities on a genome-scale. Curr Opin Sys Biol 26:46–57. doi:10.1016/j.coisb.2021.04.001.

[47] Dukovski I, Bajić D, Chacón JM, Quintin M, Vila JCC, Sulheim S, Pacheco AR, Bernstein DB, Riehl WJ, Korolev KS, Sanchez A, Harcombe WR, Segrè D. 2021. A metabolic modeling platform for the computation of microbial ecosystems in time and space (COMETS). Nat Protoc 16:5030–5082. doi:10.1038/s41596-021-00593-3. 34635859 PMC10824140

[48] Lieberman TD. 2018. Seven billion microcosms: evolution within human microbiomes. mSystems 3:e00171-17. doi:10.1128/mSystems.00171-17. 29629416 PMC5881023

[49] Bajić D, Vila JCC, Blount ZD, Sánchez A. 2018. On the deformability of an empirical fitness landscape by microbial evolution. Proc Natl Acad Sci U S A 115:11286–11291. doi:10.1073/pnas.1808485115. 30322921 PMC6217403

[50] Carey MA, Medlock GL, Stolarczyk M, Petri WA, Guler JL, Papin JA. 2019. Comparative analyses of parasites with a comprehensive database of genome-scale metabolic models. bioRxiv doi:10.1101/772467. PMC8901074 35196325

[51] Goldford JE, Lu N, Bajić D, Estrela S, Tikhonov M, Sanchez-Gorostiaga A, Segrè D, Mehta P, Sanchez A. 2018. Emergent simplicity in microbial community assembly. Science 361:469–474. doi:10.1126/science.aat1168. 30072533 PMC6405290

[52] Estrela S, Vila JCC, Lu N, Bajic D, Rebolleda-Gomez M, Chang C-Y, Sanchez A. 2020. Metabolic rules of microbial community assembly. bioRxiv doi:10.1101/2020.03.09.984278.

[53] Moran J, Tikhonov M. 2021. Coarse-graining ecological dynamics in the face of unknown microscopic details. bioRxiv doi:10.1101/2021.07.17.452786.

[54] diCenzo GC, Tesi M, Pfau T, Mengoni A, Fondi M. 2020. Genome-scale metabolic reconstruction of the symbiosis between a leguminous plant and a nitrogen-fixing bacterium. Nat Commun 11:2574. doi:10.1038/s41467-020-16484-2. 32444627 PMC7244743

[55] Rodriguez PA, Rothballer M, Chowdhury SP, Nussbaumer T, Gutjahr C, Falter-Braun P. 2019. Systems biology of plant-microbiome interactions. Mol Plant 12:804–821. doi:10.1016/j.molp.2019.05.006. 31128275

[56] Ankrah NYD, Barker BE, Song J, Wu C, McMullen JG II, Douglas AE. 2021. Predicted metabolic function of the gut microbiota of Drosophila melanogaster. mSystems 6:804–821. doi:10.1128/mSystems.01369-20. PMC8269265 33947801

[57] Medlock GL, Carey MA, McDuffie DG, Mundy MB, Giallourou N, Swann JR, Kolling GL, Papin JA. 2018. Inferring metabolic mechanisms of interaction within a defined gut microbiota. Cell Syst 7:245–257.e7. doi:10.1016/j.cels.2018.08.003. 30195437 PMC6166237

[58] Lieven C, Beber ME, Olivier BG, Bergmann FT, Ataman M, Babaei P, Bartell JA, Blank LM, Chauhan S, Correia K, Diener C, Dräger A, Ebert BE, Edirisinghe JN, Faria JP, Feist AM, Fengos G, Fleming RMT, García-Jiménez B, Hatzimanikatis V, van Helvoirt W, Henry CS, Hermjakob H, Herrgård MJ, Kaafarani A, Kim HU, King Z, Klamt S, Klipp E, Koehorst JJ, König M, Lakshmanan M, Lee D-Y, Lee SY, Lee S, Lewis NE, Liu F, Ma H, Machado D, Mahadevan R, Maia P, Mardinoglu A, Medlock GL, Monk JM, Nielsen J, Nielsen LK, Nogales J, Nookaew I, Palsson BO, Papin JA, et al. 2020. MEMOTE for standardized genome-scale metabolic model testing. Nat Biotechnol 38:272–276. doi:10.1038/s41587-020-0446-y. 32123384 PMC7082222

[59] Sulheim S, Kumelj T, van Dissel D, Salehzadeh-Yazdi A, Du C, van Wezel GP, Nieselt K, Almaas E, Wentzel A, Kerkhoven EJ. 2020. Enzyme-constrained models and omics analysis of Streptomyces coelicolor reveal metabolic changes that enhance heterologous production. iScience 23:101525. doi:10.1016/j.isci.2020.101525. 32942174 PMC7501462

[60] Kinch LN, Schaeffer RD, Kryshtafovych A, Grishin NV. 2021. Target classification in the 14th round of the critical assessment of protein structure prediction (CASP14). Proteins 89:1618–1632. doi:10.1002/prot.26202. 34350630 PMC8616802

[61] Zhou N, Jiang Y, Bergquist TR, Lee AJ, Kacsoh BZ, Crocker AW, Lewis KA, Georghiou G, Nguyen HN, Hamid MN, Davis L, Dogan T, Atalay V, Rifaioglu AS, Dalkıran A, Cetin Atalay R, Zhang C, Hurto RL, Freddolino PL, Zhang Y, Bhat P, Supek F, Fernández JM, Gemovic B, Perovic VR, Davidović RS, Sumonja N, Veljkovic N, Asgari E, Mofrad MRK, Profiti G, Savojardo C, Martelli PL, Casadio R, Boecker F, Schoof H, Kahanda I, Thurlby N, McHardy AC, Renaux A, Saidi R, Gough J, Freitas AA, Antczak M, Fabris F, Wass MN, Hou J, Cheng J, Wang Z, Romero AE, et al. 2019. The CAFA challenge reports improved protein function prediction and new functional annotations for hundreds of genes through experimental screens. Genome Biol 20:244. doi:10.1186/s13059-019-1835-8. 31744546 PMC6864930

[62] Siepel A. 2019. Challenges in funding and developing genomic software: roots and remedies. Genome Biol 20:147. doi:10.1186/s13059-019-1763-7. 31358028 PMC6664559

