# Supplementary figures and images for: Enhancing Microbiome Research through Genome-Scale Metabolic Modeling
Source: mSystems. 2021 Dec 14;6(6):e00599-21. doi: 10.1128/mSystems.00599-21 (PMC8670372; doi:10.1128/mSystems.00599-21)

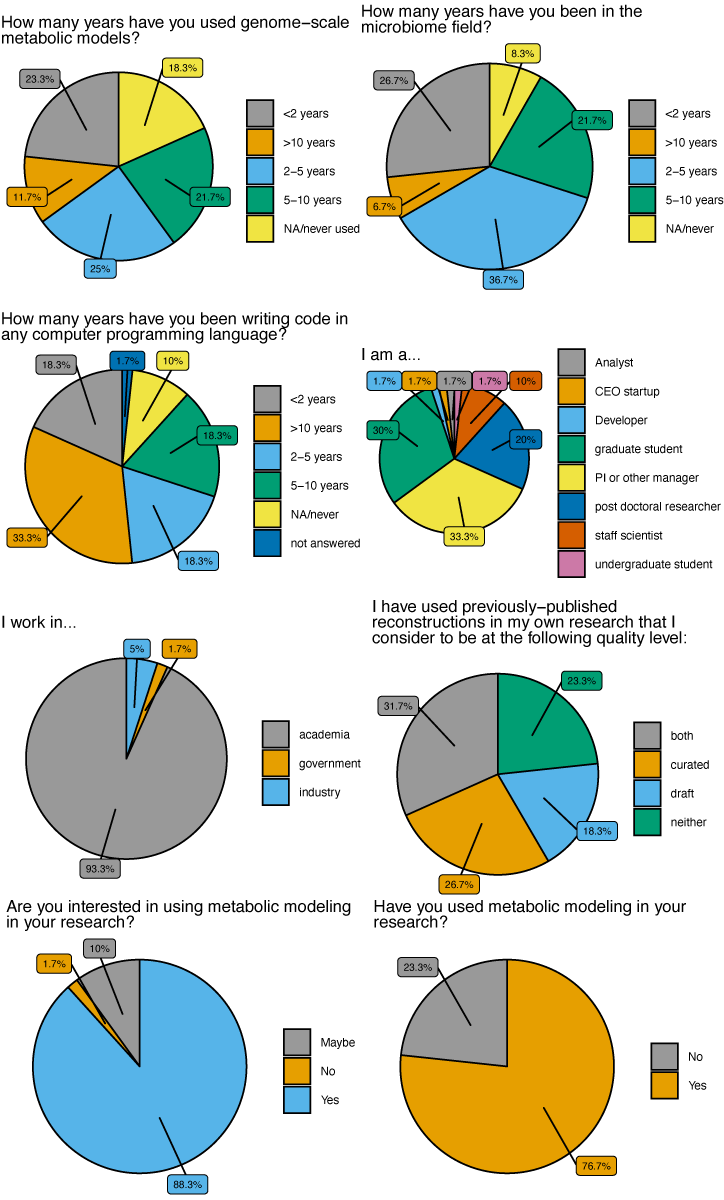

Supplement: FIG S1 [file msystems.00599-21-sf001.tif]
